# Genetic Diversity Evaluation and Population Structure Analysis of the Genus *Paphiopedilum* in Guangxi: Promoting the Selection and Breeding of New Species

**DOI:** 10.3390/ijms26178543

**Published:** 2025-09-02

**Authors:** Jianmin Tang, Kanghua Xian, Jiang Su, Li Lu, Xinru Cai, Yishan Yang, Bo Pan, Tao Ding, Xianliang Zhu, Shengfeng Chai, Rong Zou, Xiao Wei

**Affiliations:** 1 Guangxi Key Laboratory of Plant Functional Substances and Resources Sustainable Utilization, Guangxi Institute of Botany, Guilin 541006, China; 18877384841@163.com (J.T.); xkh@gxib.cn (K.X.); sj@gxib.cn (J.S.); yangyishan0113@163.com (Y.Y.); pb@gxib.cn (B.P.); dingtao@gxib.cn (T.D.); csf@gxib.cn (S.C.); 2 South China Botanical Garden, Chinese Academy of Sciences, Guangzhou 510650, China

**Keywords:** *Paphiopedilum*, genetic diversity, genetic structure, EST-SSR, conservation

## Abstract

The genus *Paphiopedilum* (Orchidaceae) has high ornamental value due to its long flowering period, brilliant flower color, and peculiar floral morphology. Guangxi is the center of ecological diversity of *Paphiopedilum*, and therefore it is urgent to conduct rescue studies on the genetic resources and genetic structure of this genus in Guangxi. In this study, the genetic diversity of 39 populations from eight *Paphiopedilum* species in Guangxi was analyzed using ten selected EST-SSR primer pairs and fluorescent PCR amplification. The results show that genetic diversity varied among species, with large differences in expected heterozygosity (*He*). The highest genetic diversity was observed in *P. barbigerum* (*I* = 0.923; *He* = 0.480), while *P. dianthum* (*I* = 0.179; *He* = 0.098) showed the lowest diversity. From the genus perspective, molecular variance analysis (AMOVA) revealed that 57% of the genetic variation occurred among populations and 43% within populations, with inter-population variation being the main source of genetic variation. From a species perspective, genetic differentiation varied, with inter-individual differentiation ranging from 79% to 95%. The percentage of molecular variance indicated that genetic variation mainly occurred among individuals, which was the main source of total variation. According to the principle of maximum likelihood, the optimal K value was determined to be 6, and 760 *Paphiopedilum* samples were divided into six subgroups. The results of this study not only identify priority populations for conservation and establish a germplasm repository to preserve existing resources, but also provide references for research on asexual reproduction, seed propagation, and hybrid breeding of *Paphiopedilum*, thereby promoting the conservation and sustainable utilization of *Paphiopedilum* germplasm resources.

## 1. Introduction

*Paphiopedilum* Pfitzer belongs to the Orchidaceae family, and its flower stems grow from the leaves, with bright and colorful flowers. There are 18 species in China, mostly distributed in Yunnan, Guizhou, and Guangxi in the southwest, which is the main distribution area of the genus *Paphiopedilum*. The majority of these species grow in limestone mountainous areas [1,2,3], mostly semi-epiphytic orchids that grow in clusters, while the other species grow on acidic sandy soil rich in organic matter, mostly terrestrial orchids that grow as single plants or scattered in tillering form. Most orchids are distributed in high-altitude areas with thin soil layers. The genus *Paphiopedilum* Pfitzer, belonging to the family Orchidaceae, produces inflorescences that arise from leaf rosettes and bears flowers with vivid and diverse colors. In China, 18 species have been recorded, mainly distributed in the southwestern provinces of Yunnan, Guizhou, and Guangxi, which constitute the primary distribution region of the genus. This area also includes several Chinese endemic species, such as *P. barbigerum* [4,5]. However, most of the more evolved Subgen. *Paphiopedilum* in the *Paphiopedilum* genus are distributed in this area, indicating that the limestone areas of Yunnan, Guizhou, and Guangxi may be the center of origin and evolution of the *Paphiopedilum* genus. The subtropical regions of China are the centers of ecological diversity of *Paphiopedilum* plants [6].

China is located in the northern margin of the species distribution of *Paphiopedilum*. The special geographical distribution has resulted in the unique resources of *Paphiopedilum*. Most of the species are geophytic or semiepiphytic. There are nine species, such as *P. armeniacum*, *P. micranthum*, *P. malipoense*, *P. concolor*, *P. bellatulum*, *P. barbigerum*, *P. emersonii*, *P. hirsutissimum*, and *P. henryanum*. Only three species are purely epiphytic, such as *P. dianthum*, *P. parishii*, and *P. villosum*. There are six species of pure geophytic orchids, such as *P. appletonianum*, *P. purpuratum*, *P. insigne*, *P. markianum*, *P. venustum*, *P. wardii*, etc. All of these species belong to the more evolved Paphiopedilum subgenus. The most primitive subgenera Brachypetala are all ground orchids or semiepiphytic orchids [7,8,9]. It can be seen that the original type of Paphiopedilum was Geophytic Orchid, which is adapted to tropical or subtropical environments because of competition pressure, and gave birth to semi-epiphytic species and then to pure epiphytic species.

*Paphiopedilum* also has high ornamental value because of its long flowering period, gorgeous flower color, and peculiar flower shape, and it is often loved by orchid enthusiasts [10,11]. The indiscriminate collection of *Paphiopedilum* species from the wild for their exotic ornamental flowers have rendered these plants endangered [12]. In addition, orchids generally grow slowly, have long reproductive cycles, and possess limited self-renewal capacity, making it difficult for populations to recover or expand naturally in the short term [13,14]. All species of the genus are listed in Figure S1 of CITES, indicating the highest level of international protection. According to the List of National Key Protected Plants newly issued by China in 2021, *Paphiopedilum* with leaves and *Paphiopedilum sclerophyllum* are classified as national second-grade protected plants, while all other species in the genus are classified as national first-grade protected plants. According to the list of national key protected wild plants issued by Guangxi Forestry Bureau (Guangxi part), there are 12 species of *Paphiopedilum* in Guangxi, 66.7% of the national species resources—nearly two-thirds. Guangxi is a possible origin and evolution center and ecological diversity center of *Paphiopedilum*. It is urgent to study the genetic resources and genetic structure of the genus *Paphiopedilum* in Guangxi, and to analyze the biological diversity resources and species evolution system of the genus *Paphiopedilum*.

Understanding genetic differentiation is essential for comprehending the intricate patterns of morphological variation both within and between species. Analyzing genetic diversity not only yields insights into the current diversity of species, but also offers valuable information about their evolutionary history and the underlying processes that have shaped this diversity [15]. Additionally, population structure analysis can provide a comprehensive view of genetic diversity levels across an entire species. By comparing rare species to common congeners, which are expected to share similar phylogenetic histories, researchers can gain critical knowledge about the extent and distribution of genetic variation within a specific genus [16,17,18].

Molecular marker technology has made great progress in the past decade. Molecular marker linkage maps of more than a dozen important crops, such as rice (*Oryza sativa* L.), wheat (*Triticum aestivum* L.), maize (*Zea mays* L.) and barley (*Hordeum vulgare* L.), have been constructed. Molecular marker technology can be used not only to draw the fingerprint of varieties and the gene linkage map of target traits, but also to study the genetic diversity of germplasm resources, and to innovate and identify germplasm resources [19]. At present, SRAP [20], RAPD [21] and ISSR [22] are used to study the genetic diversity of *Paphiopedilum* in China. Zhu [23] used SRAP molecular markers to analyze the genetic diversity of eight wild *Paphiopedilum* species in southern Guizhou. Zhu [23] used SRAP molecular markers to analyze the genetic diversity of eight wild *Paphiopedilum* species in southern Guizhou, and the results showed that the Nei′s genetic diversity index of the eight species was 0.2879, and the Shannon′s diversity index was 0.4241. Qin [24] and Li [25] used ISSR molecular markers to analyze the genetic diversity of eight *P. emersonii* populations in Guizhou and Guangxi and seven *P. micranthum* populations in Yunnan, respectively. The mean Shannon index (*I*) and expected heterozygosity (*He*) of the *P. emersonii* populations were 0.4191 and 0.2808, respectively, while for the *P. micranthum* populations, the mean *I* and *He* were 0.1943 and 0.1332, respectively. Xu [26] analyzed 190 samples from six wild *P. hirsutissimum* populations in Guangxi, Yunnan, and Guizhou using SSR molecular markers, with the six populations showing a Shannon index (*I*) of 0.8592 and *He* of 0.4387. These results indicate that genetic diversity varies significantly depending on the type of molecular marker used in *Paphiopedilum* species. Hence, marker selection in this study must carefully account for cross-species amplification, homologous and null alleles, and marker reproducibility to ensure reliable evaluation of genetic diversity. In comparison, RFLP, RAPD and ISSR technologies have been studied and applied more in major crops and horticultural crops, while AFLP and SSR are less [27]. However, some studies have shown that SSR markers have the highest number of polymorphic bands per primer combination, good repeatability, and have obvious advantages for genome-wide background selection, which is suitable for studying the genetic relationship between different germplasms and pedigree analysis between parents and offspring [28,29]. These qualities make SSRs good tools for investigating the degree and pattern of genetic variability within and between populations [30,31,32].

Therefore, for evaluation of genetic diversity among and within species of *Paphiopedilum*, we chose simple sequence repeat (SSR) markers, which are very popular markers for genetic analysis in plants, and analyze the evolutionary relationship between populations of various species within the genus *Paphiopedilum* in Guangxi [33,34]. This study aims to systematically evaluate the genetic diversity and genetic differentiation of different *Paphiopedilum* species and their populations in Guangxi, and to analyze the evolutionary relationships among these populations. By using simple sequence repeat (SSR) molecular markers, we investigated the levels of genetic diversity of various *Paphiopedilum* species and populations in Guangxi, clarified the genetic structure of each population, and revealed the extent of intra- and interspecific genetic variation. This study provides important scientific evidence for understanding the genetic diversity, genetic differentiation, and population evolutionary patterns of *Paphiopedilum*, and offers scientific guidance for the conservation and sustainable utilization of *Paphiopedilum* resources in Guangxi and the southwestern karst biodiversity center.

## 2. Results

### 2.1. Cross-Species Amplification and Microsatellite Polymorphism

As shown in Table 1, 109 alleles (*Na*) were detected by 10 pairs of primers in 760 samples, with a minimum allele number of 5, a maximum of 17, and an average allele number per locus of 10.9. The number of effective alleles (*Ne*, indicating that the more homogeneous the distribution of alleles in the population, the closer the number of alleles actually detected) was 4.162. The number of effective alleles varied from 2.144 (DL021) to 9.676 (DL032), with an average of 4.162 per locus. The Shannon Index (*I*) ranged from 0.942 (DL021) to 2.455 (DL032), with an average of 1.552. Observed heterozygosity (*Ho*) ranged from 0.127 (DL030) to 0.4 (DL020), with an average value of 0.2543. Expected heterozygosity (*He*) ranged from 0.534 (DL021) to 0.897 (DL032), with an average of 0.7117. The polymorphic information content (*PIC*) ranged from 0.474 (DL021) to 0.888 (DL032), with an average of 0.6. The average inbreeding coefficient (*F*) was 0.083, ranging from −0.07 (DL021) to −0.319 (DL034).

### 2.2. Genetic Diversity

Genetic diversity parameters of the eight *Paphiopedilum* species are summarized in Table 2. For *P. emersonii* (BHDL), the number of alleles (*Na*) ranged from 1.700 to 2.600, Shannon’s information index (*I*) ranged from 0.275 to 0.366, observed heterozygosity (*Ho*) ranged from 0.129 to 0.171, expected heterozygosity (*He*) ranged from 0.140 to 0.196, and the inbreeding coefficient (*F*) was around 0 (YZLTX = −0.043), indicating low genetic diversity. *P. dianthum* (CBDL) showed *Na* of 1.200–2.600, *I* of 0.092–0.313, *Ho* of 0.060–0.083, *He* of 0.061–0.154, and F ranging from 0.333 to 0.696, also reflecting low genetic diversity. *P. hirsutissimum* (DYDL) had *Na* of 2.500–3.300, *I* of 0.579–0.722, *Ho* of 0.289–0.402, *He* of 0.339–0.411, and mean *F* of 0.032, with some populations showing negative *F* values (NDXHJC = −0.021, MMZXKR = −0.048), indicating relatively high genetic diversity. *P. helenae* (HLDL) exhibited *Na* of 3.100–3.800, *I* of 0.775–0.961, *Ho* of 0.409–0.600, *He* of 0.409–0.537, and mean *F* of −0.053, suggesting high genetic diversity. *P. malipoense* (MLPDL) had the lowest genetic diversity, with *Na* of 1.100–2.600, *I* of 0.029–0.421, *Ho* of 0–0.195, *He* of 0.015–0.216, and *F* ranging from 0.073 to 1.000. *P. concolor* (TSDL) showed *Na* of 1.700–3.900, *I* of 0.327–0.842, *Ho* of 0.189–0.252, *He* of 0.204–0.472, and *F* of −0.016–0.483, reflecting moderate genetic diversity. *P. barbigerum* (XYDL) exhibited relatively high genetic diversity with *Na* of 3.400–4.600, *I* of 0.796–1.024, *Ho* of 0.453–0.503, *He* of 0.421–0.514, and *F* of −0.089–0.015. *P. micranthum* (YYDL) showed *Na* of 1.900–3.700, *I* of 0.282–0.630, *Ho* of 0.064–0.308, *He* of 0.160–0.333, and *F* of 0.057–0.640, indicating low to moderate genetic diversity among populations. Five populations of *P. hirsenae* had *Na* between 3.100 and 3.800, *I* between 0.775 and 0.963, *Ho* between 0.409 and 0.600 with a mean value of 0.495, *He* ranged from 0.409 to 0.537 with a mean value of 0.464, and the highest was in the LZ population with a mean *F* value of −0.053 and the presence of heterozygosity; indicating a high genetic diversity of *P. helenae* populations. Five populations of *P. malipoense* had *Na* between 1.100 and 2.600, *I* ranged from 0.029 to 0.423, *Ho* ranged from 0 to 0.195 with a mean value of 0.080, including 0 for the HJHD population, He ranged from 0.015 to 0.216 with a mean value of 0.113, and *F* ranged from 0.073 to 1 with a mean value of 0.401; indicating that the genetic diversity of *P. malipoense* populations was very low. *P. concolor*’s five populations had *Na* between 1.700 and 3.900, *I* between 0.327 and 0.842, *Ho* between 0.189 and 0.251 with a mean value of 0.228, *He* between 0.204 and 0.472 with a mean value of 0.311, and *F* with a mean value of 0.241, where TDXJHC was −0.016 and there was heterozygosity The four populations of *P. barbigerum* had *Na* between 3.400 and 3.900, *I* between 0.796 and 1.024, *Ho* between 0.453 and 0.503, with a mean of 0.487, *He* between 0.421 and 0.514, with a mean of 0.480 and *F* mean −0.001; indicating high genetic diversity in *P. barbigerum* populations. Six populations of *P. micranthum* had *Na* between 1.900 and 3.700, *I* between 0.282 and 0.603, *Ho* between 0.171 and 0.308 with a mean of 0.262, *He* between 0.160 and 0.260, and *F* ranged from 0.057 to 0.640 with a mean value of 0.179; indicating a moderate level of genetic diversity in *P. micranthum* populations.

### 2.3. Genetic Differentiation and Species Relationships

From the genus perspective, the coefficient of differentiation (*Fst*) varied from 0.410 to 0.732 across loci, with an average of 0601, indicating that 60.1% of genetic variation existed between populations, while about 39.9% of genetic variation existed within natural populations; the gene flow (*Nm*) between populations was 0.18 (Table 3). Molecular ANOVA (Table 4) is a method to measure and calculate genetic variation between haplotypes (or genotypes) by evolutionary distance. Molecular ANOVA is a method to measure and calculate genetic variation among haplotypes (or genotypes) by evolutionary distance. The analysis of molecular variance (AMOVA) indicated that 57% of the genetic variation existed among populations, and 43% existed among and within individuals, with inter-population variation being the main source of total variation in the genus. When *Nm* ≤ l, *Nm* was not sufficient to counteract the decrease in genetic diversity brought about by the effect of genetic drift, and the population genetic differentiation was large.

**Figure 1:**
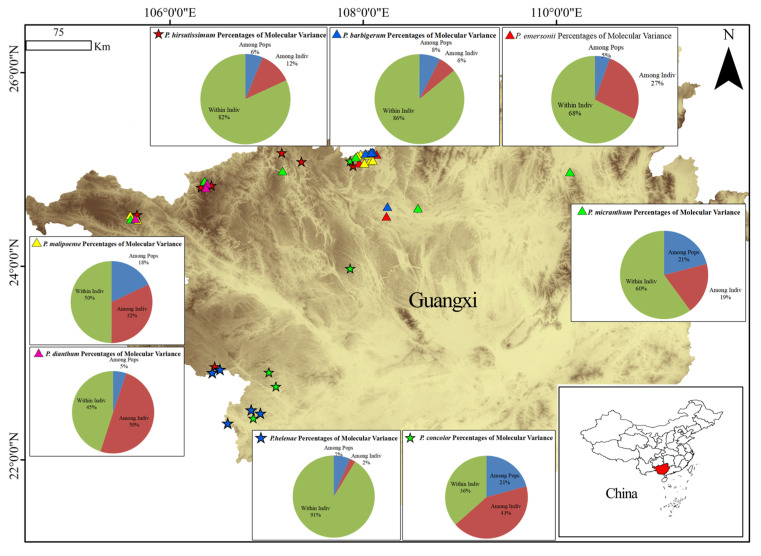
Results of hierarchical AMOVA testing from the populations.

A comparative analysis of the genetic differentiation (Figure 1) between species from the perspective of species showed that 21% of the genetic variation of *P*. *micranthum* existed among populations and 79% among individuals, and intra-group variation was the main source of variation in *P. micranthum*. According to *P. emersonii* Percentages of Molecular Variance, 5% of the genetic variation in *P. emersonii* was found among populations and 95% among individuals, and intra-population variation was the main source of variation. According to *P. malipoense* Percentages of Molecular Variance, 18% of the genetic variation in *P. malipoense* was found among populations and 88% among individuals, and intra-group variation was the main source of variation. According to *P. barbigerum* Percentages of Molecular Variance, 8% of the genetic variation in *P. barbigerum* was found among populations and 92% among individuals, and intra-group variation was the main source of variation. According to *P. dianthum* Percentages of Molecular Variance, 5% of the genetic variation in *P. dianthum* was found among populations and 95% among individuals, and intra-group variation was the main source of variation. According to *P. hirsutissimum* Percentages of Molecular Variance, 6% of the genetic variation of *P. hirsutissimum* was found among populations and 94% of the genetic variation was found among individuals, and intra-group variation was the main source of variation. According to *P. helenae* Percentages of Molecular Variance, 7% of the genetic variation of *P*. *barbigerum* was found among populations and 93% among individuals, and intra-group variation was the main source of variation. According to *P. concolor* Percentages of Molecular Variance, 21% of the genetic variation in *P. concolor* was found among populations and 79% among individuals, and intra-population variation was the main source of variation.

### 2.4. Genetic Structure and Cluster Analysis

**Figure 2:**
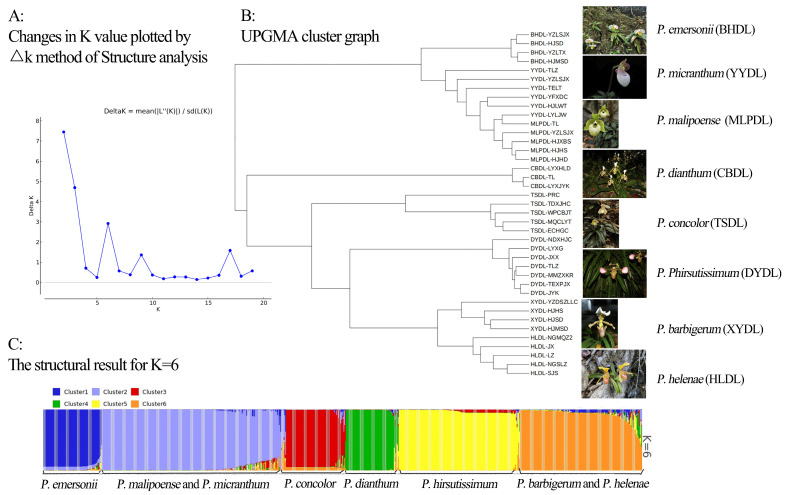
(**A**) Changes in K value plotted by AK method of Structure analysis. (**B**) UPMGA cluster graph. (**C**) The structural result for K = 6.

**Figure 3:**
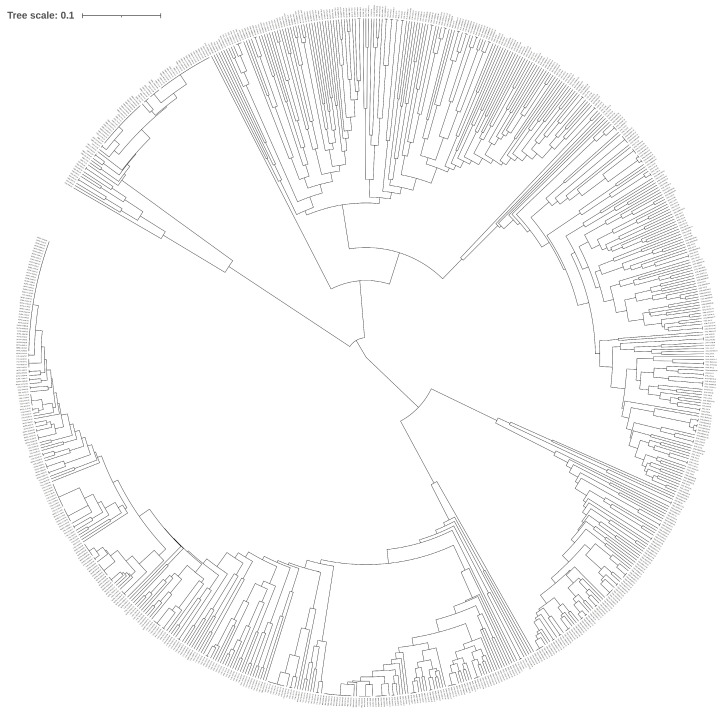
Circular cluster diagram (see Figure S1 for a clear picture).

The population structure of 760 *Paphiopedilum* samples was evaluated using 10 molecular markers. Based on the principle of maximum likelihood value, the best K value was judged to be equal to 6, and the 760 *Paphiopedilum* samples could be divided into six subpopulations. The first group (dark blue) is the *P. emersonii* population; the second group (light blue) is the *P. malipoense* and *P. micranthum* populations, and the third group (red) is the *P. concoloer* group. The fourth cluster (green) is the *P. dianthum* cluster, the fifth cluster (yellow) is the *P. hirsutissimum* cluster, and the sixth cluster (orange) is the *P. burbigerum* and *P. helenae* populations. The results are consistent with the UPMGA clustering map based on genetic distance, shown in Figure 2 and Figure 3.

### 2.5. PCoA Analysis

**Figure 4:**
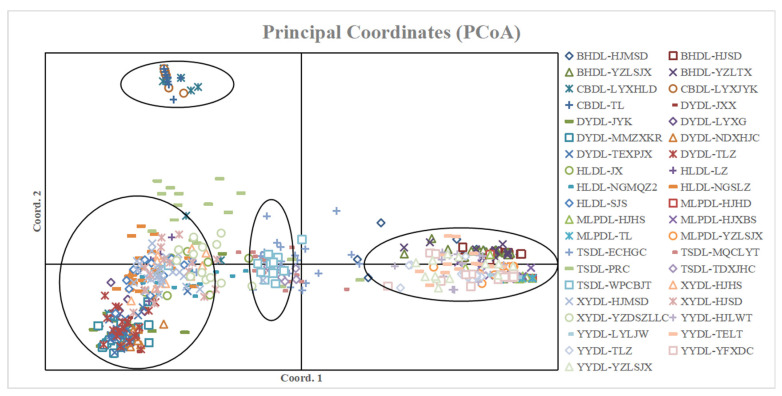
Results of principal components analysis.

Principal coordinate analysis (PCoA, Figure 4), which presents visual coordinates for studying data similarity or dissimilarity, is a non-constrained method for dimensionality reduction analysis of data and can also be used to study the similarity or dissimilarity of sample population composition. Three populations of *P. dianthum* were clustered together. The 5 populations of *P. concoloer* were more concentrated; the populations of *P. micranthum*, *P. malipoense*, and *P. emersonii* overlapped into one area; the *P. burbigerum*, *P. helenae*, and *P. hirsutissimum* clustered.

### 2.6. Genetic Distance and Geographic Distances Analysis

**Figure 5:**
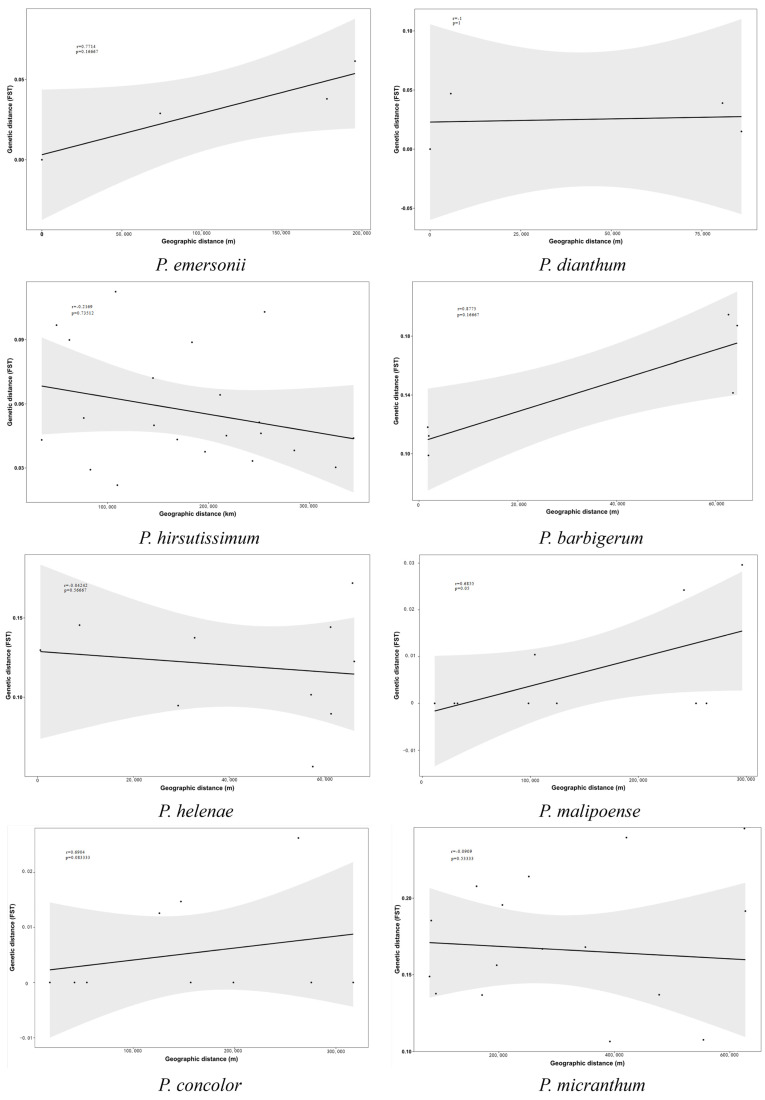
Genetic distance and geographic distances analysis.

The results of Mantel correlation analysis of geographic and genetic distances of various populations of eight species of genus *Paphiopedilum* are shown in Figure 5. Among the eight species of genus *Paphiopedilum*, only the Mantel correlation coefficient of geographic and genetic distances of *P. malipoense* was *p* ≤ 0.05, which was significantly correlated; there was no significant correlation between the genetic differentiation and geographic distribution patterns and distances of the remaining seven species of genus *Paphiopedilum*.

## 3. Discussion

Genetic diversity is a prerequisite for the survival and development of species, and its genetic level is the result of long-term evolution of the species, which can be inherited to the offspring of the population through genes, while some variations caused by developmental or environmental plasticity are not heritable [35,36]. Generally speaking, the higher the level of genetic diversity of a species, the more adaptive it is in the face of environmental changes. From an evolutionary perspective, individual organisms have limited lifespans and their contribution to evolution is realized primarily through participation in populations or population systems. Interactions among individuals within populations and gene flow between populations are key to evolutionary processes, whereas isolated individuals have limited evolutionary impact and often face lower survival probability. Therefore, genetic diversity should include both the degree of genetic variation and the distribution pattern of genetic variation (i.e., genetic structure). The distribution of genetic structure in nature is not random, but is mainly reflected by the variation in distribution form and time within and among species populations. Therefore, finding the ecological causes and genetic mechanisms that influence and constrain the genetic structure of a species can help improve the evolutionary potential of the species and its ability to resist adverse environmental conditions. The level of genetic diversity and the genetic structure maintained by an endangered species is the result of long-term evolutionary adaptation and response to the environment, and will also influence the future survival and development direction of the species [37,38,39].

Microsatellites (SSRs) are simple sequence repeats widely distributed in eukaryotic genomes. Their high polymorphism and codominant inheritance make them powerful molecular markers for studying plant genetic diversity and population structure [40,41]. In this study, we systematically assessed the genetic diversity of 760 *Paphiopedilum* samples from Guangxi by preliminarily screening and optimizing 10 pairs of SSR primers, combined with fluorescently labeled PCR amplification. For each primer, multiple replicate experiments were conducted to ensure amplification stability and data reliability, and the resulting alleles were analyzed using standardized statistical methods. The results revealed clear differences in genetic diversity among species within the genus. Both expected heterozygosity (*He*) and Shannon′s information index (*I*) indicated that *P. barbigerum* (*I* = 0.923; *He* = 0.480) had the highest level of genetic diversity, followed by *P. helenae* (*I*: 0.837; *He*: 0.464) and *P. hirsutissimum* (*I* = 0.637; *He* = 0.411). *P. concolor* (*I* = 0.519; *He* = 0.311) and *P. micranthum* (*I* = 0.502; *He* = 0.262) showed moderate levels, *P. emersonii* (*I* = 0.310; *He* = 0.172) displayed moderately low diversity, while *P. malipoense* (*I* = 0.204; *He* = 0.113) and *P. dianthum* (*I* = 0.179; *He* = 0.098) had the lowest levels. According to Nybom’s research [42], the mean values of *I* for perennial plants, widespread species, and outcrossing populations are 0.25, 0.22, and 0.27, respectively; meanwhile, the mean *He* values at the population level are 0.191 for dicotyledons, 0.20 for perennial short-lived plants, 0.28 for localized species, and 0.27 for outcrossing plants. Among them, the *He* and *I* values of *P. emersonii*, *P. micranthum*, and *P. hirsutissimum* are all lower than the results reported by Qin [23], Li [24], and Xu [24], indicating that the genetic diversity of these species in natural populations in Guangxi is limited. This may be related to local population decline, habitat fragmentation, and high levels of inbreeding. At the same time, it also reflects the significant influence of geographic distribution and environmental pressures on genetic diversity. Similarly, Cui et al. [43] analyzed the genetic diversity of 22 *Dendrobium* species using ISSR markers, reporting average *He* and *I* values of 0.133 and 0.247. For the endangered *Cypripedium tibeticum*, population *He* and *I* values were 0.3186 and 0.4843, respectively [44]. These comparisons suggest that the relatively high genetic diversity observed in species such as *P. barbigerum*, *P. helenae*, and *P. hirsutissimum* is considerably higher than that of many other endangered orchids, whereas species with low diversity such as *P. malipoense* and *P. dianthum* may face greater genetic risks due to small population sizes, habitat degradation, and self-fertilization. Nevertheless, several limitations of this study should be noted. Although the SSR primers used were effective across multiple species, they may not fully capture genome-wide diversity. Furthermore, sampling was geographically restricted, and the sample sizes for some rare species were relatively small, which could affect accuracy. In addition, the study did not explicitly examine the potential influence of environmental or ecological factors on genetic structure. Future research should incorporate higher-throughput molecular markers such as SNPs, alongside multi-regional and multi-year sampling, to provide a more comprehensive and accurate assessment of genetic diversity. Moreover, systematic comparisons with other endangered orchid species will help establish conservation priorities and guide targeted strategies for germplasm preservation.

From the genus perspective, the analysis of molecular variance showed that 57% of genetic variation existed among populations and 43% among individuals, and the variation among populations was the main source of total variation in the genus *Paphiopedilum*. A comparative analysis of genetic differentiation among species from the perspective of species showed that genetic variation existed mainly among individuals according to the percentages of Molecular Variance, where genetic variation was mainly among individuals according to *P. micranthum*, *P. emersonii*, *P. malipoense*, *P. barbigerum* and *P. malipoense*. *P. dianthum*, *P. hirsutissimum*, *P. helenae*, and *P. concolor* were 79%, 95%, 82%, 92%, 95%, 94%, 93%, and 79%, respectively, and interindividual variation was the main source of their variation. *P. micranthum* and *P. concolor* have more inter-population communication, which may be related to the wide distribution of these two species. *P. malipoense* has more distribution areas and relatively more inter-population communication, but its genetic diversity is low, and the wild resources of *P. malipoense* are drastically reduced at present, which may be related to environmental damage and factors affecting *P. malipoense* populations. The genetic diversity of *P. dianthum* is extremely low, but its populations mostly communicate among individuals, and in the original living environment, it mostly lives in symbiosis and clusters with other species. It indicated that *P. dianthum* may have outcrossing barriers in breeding and pollination, and high self-fertilization caused a sharp decrease in its own genetic diversity genes. The population variation of *P. emersonii*, *P. hirsutissimum*, and *P. helenae* is highly concentrated at the individual level, but its genetic diversity index remains relatively high, which may be related to their large population size, among which the Guangxi Yachang Orchid Plant Reserve protects the world′s largest wild population of *P. hirsutissimum* in the world.

As one of the most popular orchids in the world and also belongs to an endangered plant group, exploring the affinities between plants of the genus *Paphiopedilum* and revealing the evolutionary relationships of individual species within the genus will help the taxonomic identification of wild populations and the conservation of germplasm resources of *Paphiopedilum*. This study conducted a population structure analysis of *Paphiopedilum* in Guangxi based on microsatellite molecular markers. The results showed that the optimal K value was 6, with the samples divided into six subgroups, which were highly consistent with the phylogenetic relationships inferred from DNA barcoding. Specifically, *P. emersonii*, *P. micranthum*, and *P. malipoense* clustered together, supporting their inclusion in the subgenus *Brachypetalum*. In contrast, *P. hirsutissimum*, *P. barbigerum*, *P. concolor*, and *P. helenae* formed another cluster, all belonging to the subgenus *Paphiopedilum*, with *P. concolor* and *P. helenae* consistently grouped in both MP and Bayesian trees. *P. dianthum*, as a relatively primitive lineage within the subgenus *Paphiopedilum*, appeared as an independent branch. This concordance suggests that population structure analysis based on microsatellite markers not only reveals subgroup divisions but also reflects, to some extent, the genetic basis of phylogenetic relationships, thereby providing mutually corroborative evidence for species identification, germplasm conservation, and evolutionary studies of *Paphiopedilum*.

Genetic diversity is one of the core elements in conservation biology. Studies on the genetic diversity of *Paphiopedilum* aim to reveal the evolutionary history of the genus as well as the processes or causes underlying species endangerment. Our results indicate that populations of *Paphiopedilum* generally exhibit low genetic diversity, with some populations showing pronounced genetic differentiation and small-population characteristics, suggesting a limited capacity for natural recovery. Accordingly, conservation strategies should be tailored to specific genetic patterns: populations with relatively high genetic diversity should be prioritized as germplasm resources for establishing germplasm banks, whereas populations with low diversity and marked differentiation require artificial supplementation of propagules or ex situ conservation to mitigate the risk of inbreeding depression. When implementing population translocations or reintroductions, potential translocation risks and the risk of genetic swamping should also be carefully considered, as estimates of gene flow are model-dependent and inherently uncertain. Moreover, in population reintroduction practices, it is essential to incorporate different genetic lineages during propagation and reintroduction to enhance the genetic diversity of restored populations. Future research should include long-term genetic monitoring of key wild populations to dynamically assess the effectiveness of conservation measures, thereby ensuring the long-term preservation and sustainable utilization of *Paphiopedilum* germplasm resources. Hardy–Weinberg equilibrium (HWE) tests were conducted for each SSR locus to assess whether allele frequencies conformed to the assumptions of random mating.

## 4. Materials and Methods

### 4.1. Plant Material and Sampling Strategy

**Figure 6:**
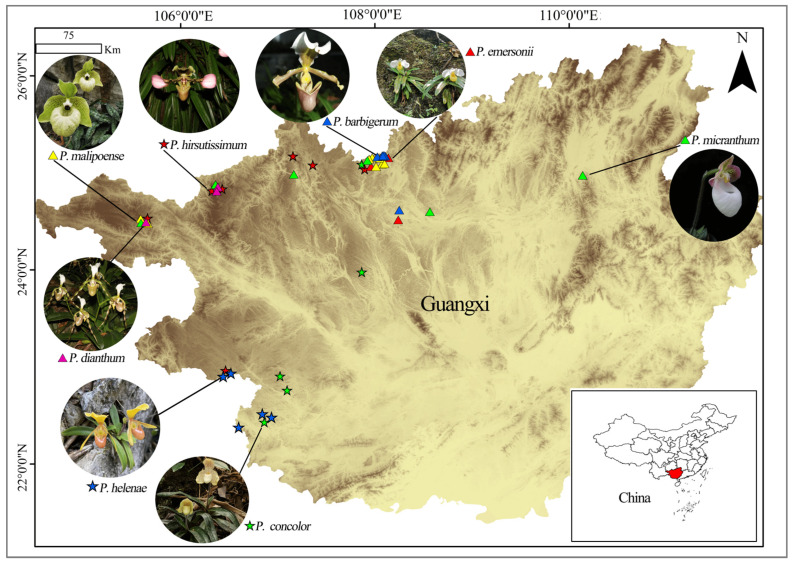
Biological characteristics, populations, and geographic locations of eight *Paphiopedilum* species in Guangxi.

In the field, species were primarily identified based on reproductive morphological characteristics, such as petal shape, labellum structure, stigma features, and peduncle length, to minimize misidentification. In addition, the collected specimens underwent rigorous taxonomic verification, including consultation of authoritative literature, reference to Flora of China and relevant monographs, and comparison with standard specimens preserved in botanical gardens or herbaria. We collected 760 samples from 39 populations of 8 species in Guangxi (there are 12 species in Guangxi, but we only collected 8 species, because the population of the remaining 4 species was small or could not be found during the collection process, so genetic testing was not conducted this time); The sample collection location, latitude and longitude, elevation and population collection number were recorded (Figure 6 and Table 5). The collected samples were packed into molecular bags and placed in sealed silica gel bags for drying and preservation. The leaf material was lyophilized and stored at −20 °C prior to DNA isolation.

### 4.2. Primer Development

Nucleic acid was extracted from the samples by using the automated workstation supported by the magnetic bead method plant genome extraction kit, and the DNA quality was detected by gel electrophoresis: concentration ≥30 ng/μL. Refer to the literature [45] for EST-SSR primer information, count the size of primer amplification fragments, group the primers reasonably, use different fluorescence to label the 5′ end of the forward primer for the same group of detected SSR sites, and synthesize the conventional primer with the reverse primer; In the marker screening and development experiment, the adapter sequence was added to the 5′ end of the forward primer, the conventional primer was synthesized by the reverse primer, and the adapter primers labeled with different fluorescent groups at the 5′ end were synthesized to improve the detection efficiency and ensure the accuracy of the results. According to the conserved sequence of microsatellite, specific primers were designed and fluorescent groups were added to carry out fluorescent PCR amplification, and the amplification products with fluorescent signals were detected by 3730 capillary fluorescent electrophoresis. Fragments with different numbers of repeat units have peak patterns with different positions. Different alleles are judged according to the reading of the peak map.

### 4.3. Microsatellite Analyses

DNA was extracted using the Plant DNA Extraction Kit based on the Merck magnetic bead method (Merck/Sigma-Aldrich, Darmstadt Germany). DNA quality was assessed by 1% agarose gel electrophoresis, and DNA concentration and purity were measured using a Nanodrop 2000 spectrophotometer (Thermo Fisher Scientific, Waltham, MA, USA). DNA samples that passed quality control were stored at −20 °C for subsequent experiments. The PCR amplification system consisted of 2×Taq PCR Master Mix 5.0 μL, DNA 1.0 μL, SSR primer 1.0 μL, and ddH_2_O 3.0 μL, with a total volume of 10 μL. DNA amplification was performed on a Veriti 384 PCR system (Thermo Fisher Scientific, USA) using the following program: initial denaturation at 95 °C for 5 min; annealing for 30 s; extension at 72 °C for 30 s; 25 cycles of denaturation at 95 °C for 30 s, annealing at 52 °C for 30 s, and extension at 72 °C for 30 s. Add fluorescent PCR products diluted to a uniform concentration to the upper plate, run the denaturation program (95 °C, 3 min), and cool immediately after denaturation is completed; Run the SSR sample analysis detection program on ABI 3730xl (Thermo Fisher Scientific, Waltham, MA, USA). We prioritized primers that produced clear and distinguishable allele peaks and well-resolved electrophoretic patterns in the samples, ensuring that the amplification products were reproducible and reliable, while exhibiting high polymorphism for subsequent population genetic analyses. Among the 48 primers screened from the literature, 10 primers with clear and suitable amplification patterns were ultimately selected after validation. Through primer synthesis and screening, 48 pairs of primers were obtained from SSR literature related to *Paphiopedilum*, and 16 samples were screened for primer validation. A total of 10 pairs of amplified primers with good peak patterns were selected (Table 6).

### 4.4. Data Analysis

The Genetic diversity indices of EST-SSR loci and population were calculated by GenAlEx version 6.501 software. Included observed allele (*Na*), effective allele (*Ne*), Shannon index (*I*), polymorphic information index (*PIC*), observed heterozygosity (*Ho*), expected heterozygosity (*He*) and inbreeding coefficient (*Fis*). The genetic distance of each population was calculated by PowerMarker 3.51 software. The UPGMA method was used for cluster analysis, and the circular cluster diagram was drawn. STRUCTURE 2.3.4 was used to analyze the population structure of 760 *Paphiopedilum* resources, setting K = 1~20, Burn-in cycle as 10,000, MCMC (MarkovChain Monte Carlo) as 100,000, each K value was run 20 times. The online tool STRUCTURE HARVESTER was used to calculate the best ΔK value (that is, the best population stratification). Plots are made based on the best K results. The results of structural analysis were plotted with CLUMMP1.1.2 and DISTRUCT 1.1 software. Based on the population genetic structure results, variation within and among populations was calculated and tested using GenAlEx 6.501, with statistical significance assessed via permutation tests (999 random permutations). Genetic differentiation coefficient (*Fst*) and gene flow (*Nm*) were calculated. Gene flow (*Nm*) was calculated according to the formula of Wright (1931) [46]: *Nm* = 0.25 (1 − *Fst*)/*Fst*.

## 5. Conclusions

The genetic diversity of the genus *Paphiopedilum* is relatively variable among the species, and genetic diversity varies considerably among the different species. The rankings according to the size of genetic diversity are as follows: *P. barbigerum* > *P. helenae* > *P. hirsutissimum* > *P. concolor* > *P. micranthum* > *P. emersonii* > *P. malipoense* > *P. dianthum*. Among them, the species with the highest genetic diversity were *P. barbigerum* (*He* = 0.480), followed by *P. helenae* (*He* = 0.464) and *P. hirsutissimum* (*He* = 0.411), and lastly, in order, *P. concolor* (*He* = 0.311), *P. micranthum* (*He* = 0.262), *P. emersonii* (*He* = 0.172), *P. malipoense* (*He* = 0.113), and *P. dianthum* (*He* = 0.098) were the least genetically diverse species. Genetic variation was mainly found among individuals in the order *P. emersonii* (95%) = *P. dianthum* (95%), *P. hirsutissimum* (94%), *P. helena* (93%), *P. barbigerum* (92%), *P. malipoense* (82%), and *P. micranthum* (79%) = *P. concolor* (79%). The detection of genetic diversity not only identifies conservation priority populations and establishes a germplasm bank to conserve existing resources, but also contributes to the conservation and sustainable utilization of the germplasm resources of *Paphiopedilum*. Based on the results of the microsatellite (SSR) analysis, population structure assessment, and phylogenetic relationships in this study, the following conclusions can be drawn: First, although some species, such as *P. barbigerum*, *P. helenae*, and *P. hirsutissimum*, exhibit relatively high genetic diversity, the overall genetic diversity of wild *Paphiopedilum* populations in Guangxi is limited. Some rare species, such as *P. malipoense* and *P. dianthum*, show notably low genetic diversity and a strong tendency toward self-fertilization. Second, population structure analysis indicates that genetic variation within each species is primarily concentrated among individuals, while a certain degree of differentiation exists between populations, which is associated with the species′ geographic distribution, habitat fragmentation, and population size.

## Figures and Tables

**Table 1:** Polymorphism of ten pairs of EST-SSR primers.

Locus	*Na*	*Ne*	*I*	*Ho*	*He*	*F*	*PIC*	Signif
DL014	5	2.68	1.133	0.172	0.627	0.726	0.555	***
DL020	17	5.545	2.049	0.469	0.82	0.428	0.802	***
DL021	6	2.144	0.942	0.23	0.534	0.568	0.474	***
DL023	17	4.011	1.728	0.182	0.751	0.757	0.72	***
DL030	6	2.536	1.074	0.127	0.606	0.79	0.531	***
DL032	17	9.676	2.455	0.42	0.897	0.532	0.888	***
DL034	14	3.242	1.462	0.166	0.692	0.76	0.644	***
DL036	6	3.385	1.341	0.153	0.705	0.783	0.656	***
DL039	15	4.563	1.96	0.357	0.781	0.543	0.763	***
DL040	6	3.38	1.372	0.267	0.704	0.62	0.657	***
Mean	10.9	4.116	1.552	0.254	0.712	0.651	0.669	
St Dev	5.466	2.197	0.485	0.121	0.107	0.129		

Note: *Na*: alleles observed; *Ne*: effective allele; *I*: Shannon index; *Ho*: observed heterozygosity; *He*: expected heterozygosity; *F*: Fixed index, an index to evaluate the degree of deviation between actual observed values and theoretical values, *PIC*: polymorphic information index; Signif: significant (ns means not significant, i.e., the population conforms to HWE; *** indicates significant difference, *p* < 0.001).

**Table 2:** Genetic diversity values and inbreeding coefficient *F* at the population level in 39 populations of 8 species.

Species	Pop	*Na*	*Ne*	*I*	*Ho*	*He*	*F*
*P. emersonii*	BHDL-HJMSD	2.600	1.281	0.360	0.148	0.186	0.202
	BHDL-HJSD	1.700	1.242	0.240	0.129	0.140	0.023
	BHDL-YZLSJX	2.500	1.441	0.366	0.181	0.196	0.054
	BHDL-YZLTX	1.900	1.270	0.275	0.171	0.166	−0.043
	Mean	2.175	1.308	0.310	0.157	0.172	0.059
*P. dianthum*	CBDL-LYXHLD	2.600	1.197	0.313	0.060	0.154	0.696
	CBDL-LYXJYK	1.200	1.112	0.092	0.063	0.061	0.367
	CBDL-TL	1.400	1.124	0.132	0.083	0.079	−0.064
	Mean	1.733	1.144	0.179	0.069	0.098	0.333
*P. hirsutissimum*	DYDL-JXX	2.500	1.730	0.600	0.330	0.349	0.087
	DYDL-JYK	3.100	1.665	0.640	0.289	0.349	0.134
	DYDL-LYXG	2.900	1.736	0.634	0.353	0.359	0.029
	DYDL-MMZXKR	2.700	1.745	0.597	0.351	0.348	−0.048
	DYDL-NDXHJC	2.600	1.895	0.604	0.331	0.339	−0.021
	DYDL-TEXPJX	3.300	1.793	0.661	0.326	0.362	0.030
	DYDL-TLZ	2.800	1.981	0.722	0.402	0.411	0.017
	Mean	2.843	1.792	0.637	0.340	0.360	0.032
*P. helenae*	HLDL-JX	3.400	2.513	0.961	0.550	0.537	−0.044
	HLDL-LZ	3.100	2.267	0.876	0.600	0.507	−0.143
	HLDL-NGMQZ2	3.300	2.210	0.789	0.490	0.442	−0.114
	HLDL-NGSLZ	3.800	2.057	0.785	0.409	0.425	0.007
	HLDL-SJS	3.500	2.274	0.775	0.425	0.409	0.030
	Mean	3.420	2.264	0.837	0.495	0.464	−0.053
*P. malipoense*	MLPDL-HJHD	1.100	1.018	0.029	0.000	0.015	1.000
	MLPDL-HJHS	1.700	1.158	0.174	0.043	0.099	0.504
	MLPDL-HJXBS	2.100	1.241	0.274	0.086	0.152	0.318
	MLPDL-TL	1.200	1.147	0.123	0.078	0.085	0.112
	MLPDL-YZLSJX	2.600	1.515	0.421	0.195	0.216	0.073
	Mean	1.740	1.216	0.204	0.080	0.113	0.401
*P. concolor*	TSDL-ECHGC	3.900	1.783	0.671	0.220	0.340	0.402
	TSDL-MQCLYT	3.100	1.727	0.513	0.189	0.252	0.291
	TSDL-PRC	3.400	2.132	0.842	0.252	0.472	0.483
	TSDL-TDXJHC	1.700	1.453	0.327	0.230	0.204	−0.016
	TSDL-WPCBJT	2.500	1.624	0.519	0.251	0.287	0.241
	Mean	2.920	1.744	0.574	0.228	0.311	0.280
*P. barbigerum*	XYDL-HJHS	3.400	2.215	0.796	0.503	0.421	−0.089
	XYDL-HJMSD	4.600	2.799	1.024	0.493	0.514	0.015
	XYDL-HJSD	3.600	2.568	0.913	0.453	0.484	0.058
	XYDL-YZDSZLLC	3.900	2.583	0.960	0.498	0.501	0.011
	Mean	3.875	2.541	0.923	0.487	0.480	−0.001
*P. micranthum*	YYDL-HJLWT	3.700	1.773	0.586	0.252	0.281	0.115
	YYDL-LYLJW	1.900	1.362	0.282	0.171	0.160	0.057
	YYDL-TELT	3.100	1.888	0.630	0.308	0.333	0.063
	YYDL-TLZ	2.600	1.506	0.404	0.064	0.204	0.640
	YYDL-YFXDC	2.700	1.790	0.541	0.262	0.285	0.124
	YYDL-YZLSJX	2.600	1.838	0.570	0.286	0.311	0.074
	Mean	2.767	1.693	0.502	0.224	0.262	0.179

*Na*: observed alleles; *Ne*: effective allele; *I*: Shannon Information Index; *Ho*: observed heterozygosity; *He*: expected heterozygosity; *F*: Fixed index.

**Table 3:** Inbreeding coefficients and gene flow of 10 primer pairs.

Locus	*Fis*	*Fit*	*Fst*	*Nm*
DL014	−0.004	0.711	0.712	0.101
DL020	−0.024	0.395	0.409	0.361
DL021	−0.070	0.530	0.560	0.196
DL023	0.028	0.729	0.721	0.097
DL030	0.188	0.782	0.732	0.091
DL032	0.196	0.526	0.410	0.359
DL034	0.319	0.766	0.657	0.130
DL036	0.169	0.759	0.710	0.102
DL039	0.051	0.525	0.500	0.250
DL040	−0.028	0.588	0.599	0.167
Mean	0.083	0.631	0.601	0.186
SE	0.040	0.043	0.040	0.033

Note: *Fis:* inbreeding coefficient within a population, *Fit:* overall inbreeding coefficient, *Fst*: genetic differentiation coefficient, *Nm*: gene flow (*Nm* = 0.25 (1 − *Fst*)/*Fst*).

**Table 4:** Molecular Analysis of Variance (AMOVA).

Source	df	SS	MS	Est. Var.	%
Among Pops	38	3169.945	83.420	2.097	57%
Among Indiv	721	1394.600	1.934	0.354	10%
Within Indiv	760	932.500	1.227	1.227	33%
Total	1519	5497.044	5497.045	3.678	100%

Source: source of variation; df: Freedom of freedom; SS: total variance; MS: mean square error; Est. Var.: estimated variance; %: variation percentage; Among Pops: Gene flow between populations; Among Indiv: Gene flow among individuals of a species; Within Indiv: within a group of individuals.

**Table 5:** Populations and geographic locations of eight *Paphiopedilum* species.

Species	P. Code	Location	No.	E	N	H
*P. emersonii*	BHDL-YZLSJX	Yizhou District, Hechi City, Guangxi	20	108°34′	24°36′	437
	BHDL-HJMSD	Huanjiang County, Hechi City, Guangxi	20	108°03′	25°09′	577
	BHDL-YZLTX	Yizhou District, Hechi City, Guangxi	20	108°14′	24°30′	218
	BHDL-HJSD	Huanjiang County, Hechi City, Guangxi	14	108°02′	25°09′	592
*P. micranthum*	YYDL-LYLJW	Leye County, Baise City, Guangxi	28	106°22′	24°50′	640
	YYDL-YZLSJX	Yizhou District, Hechi City, Guangxi	14	108°34′	24°36′	285
	YYDL-TELT	Tian′e County, Hechi City, Guangxi	26	107°09′	24°59′	870
	YYDL-HJMLT	Huanjiang County, Hechi City, Guangxi	29	108°02′	25°08′	977
	YYDL-YFXDX	Yongfu County, Guilin City, Guangxi	16	110°08′	24°58′	363
	YYDL-TLZ	Tianlin County, Baise City, Guangxi	15	105°38′	24°30′	1122
*P. barbigerum*	XYDL-YZDSZLLC	Yizhou District, Hechi City, Guangxi	16	108°15′	24°37′	268
	XYDL-HJHS	Huanjiang County, Hechi City, Guangxi	30	108°03′	25°09′	533
	XYDL-HJSD	Huanjiang County, Hechi City, Guangxi	17	108°02′	25°09′	541
	XYDL-HJMSD	Huanjiang County, Hechi City, Guangxi	10	108°03′	25°09′	537
*P. malipoense*	MLPDL-YZLSJX	Yizhou District, Hechi City, Guangxi	15	108°34′	24°36′	285
	MLPDL-HJHS	Huanjiang County, Hechi City, Guangxi	30	108°03′	25°09′	398
	MLPDL-HJHD	Huanjiang County, Hechi City, Guangxi	24	108°03′	25°08′	377
	MLPDL-HJXBS	Huanjiang County, Hechi City, Guangxi	20	107°56′	25°08′	709
	MLPDL-TL	Tianlin County, Baise City, Guangxi	9	105°38′	24°30′	1122
*P. concolor*	TSDL-PRC	Zuozhou District, Chongzuo City, Guangxi	25	107°57′	25°06′	732
	TSDL-WPCBJT	Dahua County, Hechi City, Guangxi	20	107°51′	23°58′	567
	TSDL-TDXJHC	Tiandeng County, Chongzuo City, Guangxi	9	107°01′	22°54′	384
	TSDL-ECHGC	Daxin County, Chongzuo City, Guangxi	33	107°05′	22°45′	356
	TSDL-MQCLYT	Longzhou County, Chongzuo City, Guangxi	22	106°53′	22°26′	404
*P. dianthum*	CBDL-LYXJYK	Leye County, Baise City, Guangxi	19	106°24′	24°50′	400
	CBDL-LYXHJD	Leye County, Baise City, Guangxi	27	106°22′	24°48′	700
	CBDL-TL	Tianlin County, Baise City, Guangxi	20	105°38′	24°30′	1110
*P. hirsutissimum*	DYDL-PEXPJX	Tian′e County, Hechi City, Guangxi	29	107°08′	25°11′	375
	DYDL-JYK	Leye County, Baise City, Guangxi	23	106°24′	24°50′	400
	DYDL-LYXG	Leye County, Baise City, Guangxi	15	106°19′	24°48′	820
	DYDL-NDXHJC	Nandan County, Hechi City, Guangxi	20	107°21′	25°05′	386
	DYDL-MMZXKR	Huanjiang County, Hechi City, Guangxi	30	107°57′	25°06′	732
	DYDL-JXX	Jinxi City, Guangxi	10	106°29′	22°55′	750
	DYDL-TLZ	Tianlin County, Baise City, Guangxi	24	105°38′	24°30′	1122
*P. helenae*	HLDL-NGSLZ	Longzhou County, Chongzuo City, Guangxi	22	106°50′	22°31′	547
	HLDL-LZ	Longzhou County, Chongzuo City, Guangxi	11	106°35′	22°22′	429
	HLDL-NGMQZ2	Longzhou County, Chongzuo City, Guangxi	19	106°54′	22°27′	430
	HLDL-JX	Jinxi City, Guangxi	7	106°29′	22°55′	790
	HLDL-SJS	Jinxi City, Guangxi	12	106°28′	22°54′	700

No. = sample size; P. Code = Population code; E = East longitude; N = North latitude; H = altitude.

**Table 6:** Information on EST-SSR primers.

Locus	Repeat Motif	Primer Sequence (F)	Primer Sequence (R)	Ta (°C)	GenBank No.
DL014	(CTC)6	TTCCTTCCCTACCCTTTCCA	CAGCGGTGTCGTTGATGTT	60	MG333725
DL020	(GCC)6	GGCCAAGTACATGCACCCAT	TTCCCACCTCGGTTATGCAC	60	MG333700
DL021	(CAG)6	GCAAATCCATTCAGCCCTGC	CGACATGGTCTGAGAGGAGC	60	MG333701
DL023	(AGA)6	CTTGGGACTCTTTCCTCGGC	CAGCACCTCTTCGCGTAAGA	60	MG333703
DL030	(CCG)6	CAGGTTGACAGCAATGTCGC	GCCGCAGCTTTTCGGATAAG	60	MG333710
DL032	(AAAC)5	AGCGTGTTTGGACTAGAGCA	TCGGGGATGCACATGGAAAA	60	MG333712
DL034	(CGG)6	GGGTGGGGAGAGTAGGAGTT	GCCACAACTTGTTTTCCCGG	60	MG333714
DL036	(CGT)6	CCACGTGTGACAGAATCCCA	GGCTCCCGACGAGGAATTAC	60	MG333716
DL039	(ATC)6	CCACCAGCTTTCATATCCTCCA	GCCCATGCTGTGCAAAAAGA	60	MG333719
DL040	(TCT)6	AAGAAGTGGCTTCCATGGCA	GCAAAACCAAGGTGTCGTCC	60	MG333720

## Data Availability

Data will be made available on request.

## Supplementary Materials

The following supporting information can be downloaded at: https://www.mdpi.com/article/10.3390/ijms26178543/s1.
